# Formation, Photophysics, and Photochemistry of Anionic Lanthanide(III) Mono- and Bisporphyrins

**DOI:** 10.3390/molecules24071309

**Published:** 2019-04-03

**Authors:** Muhammad Imran, Melitta P. Kiss, Zsolt Valicsek, Ottó Horváth

**Affiliations:** 1 Department of Chemistry, Baghdad-Ul-Jadeed Campus, The Islamia University of Bahawalpur, Bahawalpur 63100, Pakistan; muhammadimranum@yahoo.com; 2 Department of General and Inorganic Chemistry, Institute of Chemistry, Faculty of Engineering, University of Pannonia, P.O.B. 158, H-8201 Veszprém, Hungary; melitta.kiss@audi.hu (M.P.K.); valicsek@almos.uni-pannon.hu (Z.V.)

**Keywords:** out-of-plane metalloporphyrins, lanthanide(III) ions, tail-to-tail oligomerization, ligand-to-metal-charge transfer, charge separation, fluorescence, photolysis, samarium, europium, gadolinium

## Abstract

Since water-soluble porphyrin complexes of lanthanides(III) have proved to be promising for medical applications (e.g., luminescence imaging, photodynamic therapy, and theranostics), the investigation of the formation, photophysical, and photochemical properties of such coordination compounds provides useful pieces of information for their potential usage. Steady-state and time-resolved fluorometry, UV–Vis absorption spectroscopy, and continuous-wave photolysis were utilized for this purpose. 5,10,15,20-Tetrakis(4-sulfonatophenyl)porphyrin formed mono- and bisporphyrin complexes with samarium(III), europium(III), and gadolinium(III) as representatives in the middle of the lanthanide series. The special photoinduced behavior of these compounds was mostly determined by the position of the metal center, which was located out of the ligand plane, thus distorting it. Besides, the photochemical and, especially, photophysical features of the corresponding mono- and bisporphyrin complexes were similar because, in the latter species, two monoporphyrins were connected by a weak metal bridge between the peripheral sulfonato substituents (tail-to-tail dimerization). The formation of these coordination compounds and the transformation reactions between the mono- and bisporphyrins were rather slow in the dark at room temperature. These processes were accelerated by visible irradiation. However, dissociation and, especially, redox degradation were the main photoreactions in these systems, although with low quantum yields. Additionally, depending on the excitation wavelength, new types of photoproducts were also detected.

## 1. Introduction

Metalloporphyrins and their derivatives represent a very important group of compounds that play key roles in several natural and artificial systems, due to their peculiar light-absorption and -utilization, coordination, and redox features. Chlorophylls are the main antenna units in green plants for harvesting solar radiation in photosynthesis [1]. Hemoglobin and myoglobin are responsible for oxygen transport in blood [2], while iron-porphyrin-based cytochromes are important electron relays and redox catalysts [3].

Synthetic metalloporphyrins have been used, for example, for medical treatments (e.g., photodynamic therapy (PDT) [4]), photocatalytic systems utilizing visible light to generate hydrogen and other fuels [5,6,7,8], and cleavage or release of axially coordinated NO_×_ ligands [9,10,11].

**Figure 1:**
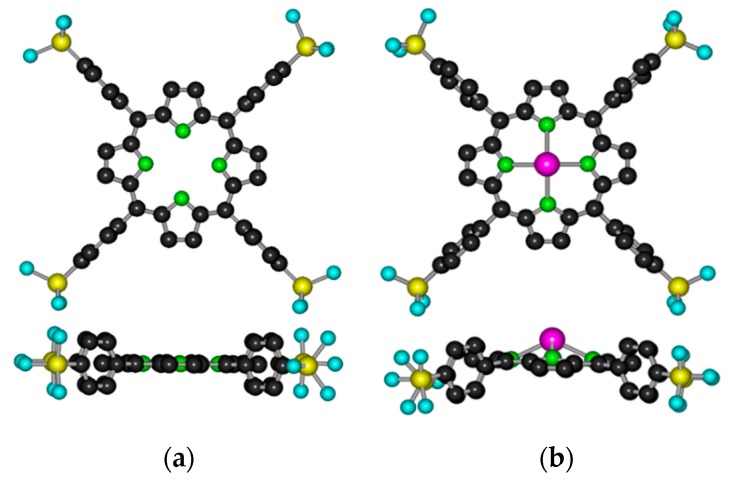
Top- and side-views of (**a**) the free-base 5,10,15,20-tetrakis(4-sulfonatophenyl)porphyrin and (**b**) its (out-of-plane) lanthanide(III) complex. (The hydrogen atoms are omitted for clarity.)

In aqueous media, various porphyrins with suitable ionic groups have been applied. Cationic metalloporphyrins (with methylpyridinium substituents) could be successfully utilized for photocatalytic electron transfer with cobalt(III) [12], manganese(III) [13], and nickel(II) [14] centers [15]. For anionic derivatives, mostly carboxylate and sulfonate substituents have been used. Since the coordination bond between carboxylate and transition metal ions may be sensitive to photoinduced ligand-to-metal (LMCT) processes, in several cases, sulfonation was utilized to transform organic ligands and macrocycles into anionic derivatives for photophysical and -chemical applications in various aqueous systems [16,17,18,19,20,21]. Figure 1a shows the structure of 5,10,15,20-tetrakis(4-sulfonatophenyl)porphyrin (H_2_TSPP^4−^), a typical example of these anionic ligands.

Lanthanides (abbreviated as Ln) are a series of elements with atomic numbers 57–71, located in the first row of the *f*-block in the periodic table. Lanthanides usually form cations with a +3 oxidation state, and the radius of the Ln^3+^ ions decreases from left to right in the periodic table; this is the well-known lanthanide contraction [22]. The peculiar electronic configuration of lanthanide(III) ions ([Xe]4*f^n^*, *n* = 1–14) results in special photophysical properties [23]. Their absorption spectra are line-like and originate from *f*–*f* transitions [24]. The lanthanide ions, except for Lu^3+^ and Gd^3+^, show luminescence in the UV and NIR region. Lu^3+^, being completely filled, and Gd^3+^, being half-filled, are not luminescent. Although lanthanide(III) ions absorb very weakly in the visible range, their emissions can be sensitized by absorption of organic ligands which transfer energy to the metal center [25,26], providing appropriate compounds for medical diagnostic applications [27,28,29].

Lanthanide(III) ions, being hard Lewis acids, prefer to bind hard bases possessing oxygen-donor groups [22,30]. The coordination number of lanthanides may be even 12, but 8 and 9 are most common, and they are advantageous for the formation of sandwich-type complexes with various macrocycles, producing cubic or square antiprism polyhedra [31,32]. In the absence of a hard basis, Ln^3+^ ions can be coordinated to other types of ligands, such as N-donor porphyrins [33]. This process, however, is very slow due to the high activation energy.

In complexes with porphyrins, also due to their large ionic radii, lanthanide(III) ions are positioned above the ligand plane and form sitting-atop (SAT) or out-of-plane (OOP) metalloporphyrins (Figure 1b) [34,35,36,37,38]. In sandwich-type complexes of porphyrins, the π–π interactions between the macrocycles can result in interesting electronic, steric, photochemical, and, especially, photophysical features deviating from those of the corresponding monoporphyrin [39,40].

Lanthanide porphyrins have proved to very useful and promising in a broad range of application possibilities. Several studies focused on the photophysical properties of Ln(III) porphyrins ([41] and the references therein). Various emission features of Nd, Gd, Er, and Yb complexes were determined in different solvents [42]. The luminescence behavior of Gd(III) and Lu(III) porphyrins indicated their possible application in oxygen sensing [43]. Another significant utilization of the photophysical properties of such complexes is sensitization of NIR emission of the lanthanide(III) center, due to the porphyrin-based, long-lived triplet excited state [44,45]. Since NIR radiation penetrates human tissues considerably deeper than visible photons do, lanthanide porphyrins offer good possibilities in bio-imaging [46,47].

In water-soluble lanthanide oligoporphyrins, not only sandwich-type (head-to-head) complexes occur, but tail-to-tail connections between the monoporphyrin units are possible through peripheral hard-base substituents (such as carboxylate) and hard-acid Ln(III) ions [48].

The SAT or OOP water-soluble porphyrin complexes display a typical photoredox chemistry characterized by an irreversible photodegradation of the porphyrin ligand [18,20]. This special photochemical feature, due to an efficient separation of the reduced metal center and the oxidized macrocycle, following a ligand-to-metal charge transfer, was also observed for water-soluble lanthanide(III) porphyrins [15,30,37].

Since photophysical and photochemical properties of such complexes are crucial from the viewpoint of their application in aqueous systems (such as in human bodies or various in vitro procedures), the scrutiny of photoinduced properties of lanthanide(III) complexes with water-soluble porphyrins is highly desirable. Hence, continuing our systematic investigation, after studying the coordination compounds of early lanthanides with anionic H_2_TSPP^4−^ [30,38], three members in the middle of the series (i.e., Sm(III), Eu(III), and Gd(III)) were examined in this work. Our main goals were to determine the composition, structure, and formation constant of these complexes. Besides photophysical experiments (both absorption and emission spectroscopic measurements, including fluorescence life-time determinations), steady-state photochemical investigations were also carried out for the characterization of the light-induced behavior of these metalloporphyrins.

Our results clearly demonstrated that the luminescence properties of the bisporphyrins hardly deviated from those of the monomers, indicating a weak interaction between the constituting units. Besides, the photochemistry of these lanthanide(III) porphyrins involved several types of reactions (such as redox degradation, dissociation, and transformations), although the quantum yields of these processes were low. Hence, these photolytic reactions are not expected to considerably limit the applicability of these complexes either for medical, mostly diagnostic, purposes or as sensitizers in photocatalytic systems. Rather, the photoinduced redox reactions may provide opportunities for PDT, too (together with the sensitized production of singlet oxygen) [4].

## 2. Results and Discussion

### 2.1. Formation, Composition, and Absorption Spectra

The efficient light absorption of porphyrins and their metal complexes makes UV–Vis spectroscopy a suitable method to quantitatively investigate the formation, composition, and structure of water-soluble lanthanide(III) porphyrins. Since the insertion of hard-acid lanthanide(III) ions into the cavity of anionic porphyrin is a very slow process, partly due to the strong Ln(III)–H_2_O coordinative bond, the formation reaction was carried out at 60 °C. According to our previous observations [30,38], this temperature proved to be enough to overcome the kinetic barrier of the insertion. Depending on the anions in the electrolyte applied to adjust the ionic strength (to I = 0.01 M), the formation of mono- and bisporphyrin complexes occurs. Since in the visible range the absorption of lanthanides(III) is negligible compared with that of porphyrins, metal ion excess was applied in our work to study the formation of these kinetically labile complexes. The behaviors of the three lanthanide(III) ions in this study were very similar; hence, the spectral changes regarding the systems containing Gd(III) are presented to demonstrate the mutual characteristic features, as gadolinium is the most widely investigated and applied member of this triad.

**Figure 2:**
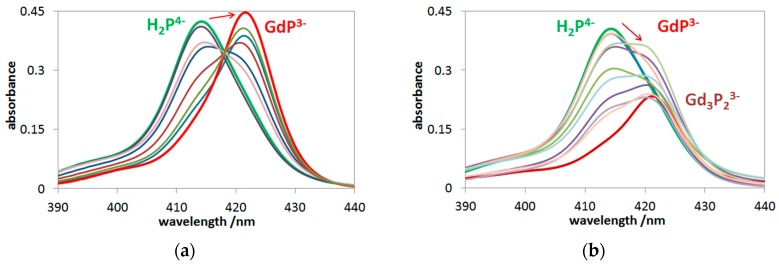
Spectrophotometric titration: (**a**) 1.3 × 10^−6^ M H_2_P^4−^ and 0–9.2 × 10^−4^ M Gd(III), 0.01 M acetate and (**b**) 1.0 × 10^−6^ M H_2_P^4−^ and 0–1 × 10^−3^ M Gd(III), 0.01 M perchlorate.

In the presence of acetate ions, which strongly coordinate to lanthanides(III) [48], only a monoporphyrin complex could form (Figure 2a), while the noncoordinating perchlorate ions allowed the formation of bisporphyrin, too (Figure 2b).

Insertion of a metal ion into the porphyrin cavity, forming an OOP complex, was manifested by the redshift of the main Soret-band, as shown in Figure 2a (from 413 to 421 nm), in accordance with the case of other OOP metallo-monoporphyrins (e.g., with Fe(II), Hg(II), Hg(I), Tl(I), and Cd(II) ions [19,49]). Besides the monoporphyrin complex, in the presence of perchlorate, the formation of bisporphyrin (Gd_3_P_2_^3−^) also took place as the broadening of the Soret-band, along with the decrease of the absorbances, compared with the case of the monoporphyrin (Figure 2b). Similar spectral changes were measured for the corresponding samarium(III) and europium(III) systems, as well as the earlier investigated lanthanum(III), cerium(III), and neodymium(III) porphyrins [15].

**Figure 3:**
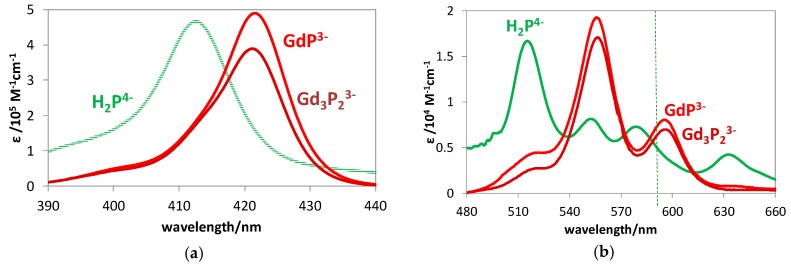
Molar absorption spectra of GdP^3−^ and Gd_3_P_2_^3−^ compared with those of the free-base porphyrin in the (**a**) Soret-range and (**b**) Q-range.

The evaluation of the data regarding the spectrophotometric titrations, described in Section 3.3, provided the stability constants and the individual molar absorption spectra of mono- and bisporphyrin complexes in both the Soret- and Q-range. Figure 3 displays the spectra of the studied gadolinium(III) porphyrins compared with those of the corresponding free base.

Table 1 summarizes the formation constants for the Sm(III), Eu(III), and Gd(III) porphyrins in these systems.

The values determined for the monoporphyrins in acetate solutions (lgβ_1:1_(Ac^−^)) were moderately higher than the corresponding ones obtained in the presence of perchlorate ions. This difference may be interpreted by the trans effect of the acetate ion as a bidentate hard-base (*O*-donor) ligand bound to the lanthanide(III) ions. It promotes the coordination of the first porphyrin (but hampers the connection of an additional one) [33].

Although the decrease of the ionic radius in the order of Sm(III) > Eu(III) > Gd(III) was rather moderate, the increase of the corresponding formation constants was similarly monotonous. This tendency can be explained by the shorter coordination bonds due to the better fit of the smaller metal ions into the porphyrin cavity. This interpretation is confirmed by the formation constants obtained earlier for the corresponding La(III), Ce(III), and Nd(III) complexes [37,51]. For example, the values for the formation of cerium(III) monoporphyrin (lgβ_1:1_(Ac^−^) = 4.00; lgβ_1:1_ = 3.60 [51]) are one order of magnitude lower than those of the europium(III) complex, in accordance with the significantly higher ionic radius of Ce(III) (114.3 pm [50]). The same tendency was valid for the lgβ_3:2_ values, even if the increase in the order of Sm(III) < Eu(III) < Gd(III) was very slight. However, compared with the value for the Ce_3_P_2_ complex (lgβ_3:2_ = 16.30), the formation constant of the Eu(III) bisporphyrin was more than one order of magnitude higher.

After the determination of the individual molar absorption spectra, analyses of the Soret- and Q-bands of the lanthanide(III) porphyrins studied were carried out by fitting Gaussian curves. The most important data obtained are shown in Table 2 (in Section 2.2), compared to the corresponding values regarding the spectrum of the free-base porphyrin.

### 2.2. Photophysics of Mono- and Bisporphyrins

**Figure 4:**
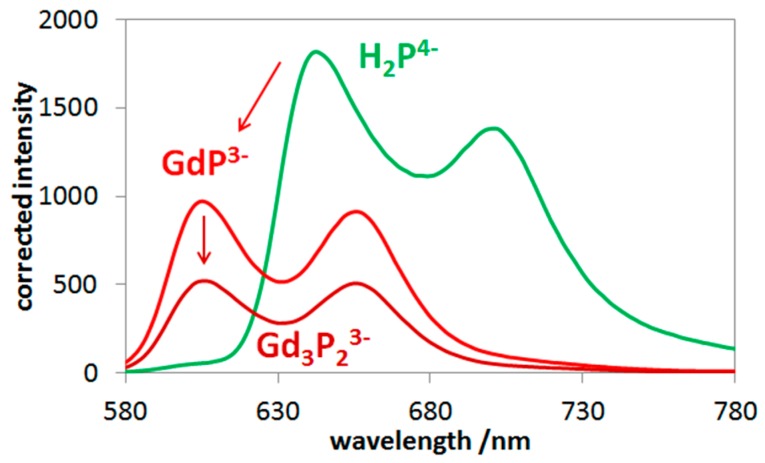
Individual S_1_-fluorescence spectra of gadolinium(III) mono- and bisporphyrin as well as the corresponding free base.

To elucidate the structure and the mode of coordination in these lanthanide complexes, particularly in the bisporphyrins, in aqueous systems, their S_1_-fluorescence properties were also investigated. Similar to the light-absorption experiments, spectrofluorimetric titrations were also carried out with these systems. The quasi-isosbestic point of the absorption spectra (see Figure 3a) was chosen (at about 417 nm) as the excitation wavelength for the elimination of disturbing absorbance changes during the titration process. From the data of these spectral series, also regarding the systems containing bisporphyrins, the individual emission spectra could be determined. Figure 4 displays the fluorescence spectra of the emitting species in the gadolinium(III)–porphyrin system. Insertion of the lanthanide(III) ion into the porphyrin cavity resulted in a significant blueshift of the fluorescence bands accompanied by a moderate change of their intensity ratio. The characteristic photophysical data of the mono- and bisporphyrins studied, compared to those of the free-base porphyrin, are summarized in Table 2.

As previously observed, the insertion of the metal center into the porphyrin cavity (in an out-of-plane position) diminished the fluorescence efficiency of the free base, due mostly to the distortion of the ligand plane and partly to electronic interactions. The fluorescence quantum yields for the Sm(III), Eu(III), and Gd(III) porphyrins investigated were considerably higher than those for the corresponding La(III) and Ce(III) complexes (e.g., 2.74 × 10^−2^ and 1.03 × 10^−2^ for the La(III) mono- and bisporphyrins, respectively) [15]. This suggests that the significantly larger ionic radius (116 and 114 pm for La^3+^ and Ce^3+^, respectively), resulting in a higher out-of-plane distance, caused a stronger (dome) distortion of the porphyrin ring. In the case of the corresponding Nd(III) porphyrins, the quantum yield values were similar to those of the Sm(III), Eu(III), and Gd(III) complexes due to the smaller difference between the ionic radii (111 pm for Nd^3+^) [15]. Interestingly, however, within the group of the lanthanide(III) ions studied in this work, the quantum yield slightly decreased with the decreasing ionic radius. This phenomenon may be interpreted by an electronic interaction with the unpaired electrons of the metal center, the number of which increased up to the half-filled subshell of Gd^3+^. The lower ionic radius, due to the shorter bond length, strengthened this effect.

Excitation of these complexes at the Soret-band led to S_1_-fluoresence following an efficient internal conversion from the second singlet excited state to the first one. The ratio of the fluorescence quantum yields obtained by Soret- and Q-band excitations gave the efficiency of the internal conversion (ϕ(IC S_2_–S_1_) = Ф(S_1_ − fluo@B)/ϕ(S_1_ − fluo@Q)).This was a considerably efficient process in the case of these porphyrins (ϕ(IC S_2_–S_1_) was about 0.7), for both the free base and the lanthanide complexes. Interestingly, while the sandwich-type (head-to-head) OOP metallo-bisporphyrins did not display any appreciable S_1_-fluorescence [40], the emission quantum yield of the Ln_3_P_2_^3−^ (tail-to-tail) complexes was about the same order of magnitude as that of the corresponding monoporphyrin. The fluorescence lifetimes were about the same value (1.94 ns) for all lanthanide porphyrins studied. This suggests that there was no significant electronic interaction between the porphyrin rings in the bisporphyrin structure. Basically, the effects of the metal center in the ligand cavity caused the change in the emission properties, compared to those of the free base. The lifetime for the S_1_-fluorescence of the free-base porphyrin was 10.0 ns (Table 2). The coordination of the lanthanide ions into the porphyrin cavity considerably diminished the emission lifetime to less than one-fifth of that of the free-base ligand. This was mainly due to the more complex and distorted structure significantly accelerating the nonradiative decay of the S_1_ excited state.

**Figure 5:**
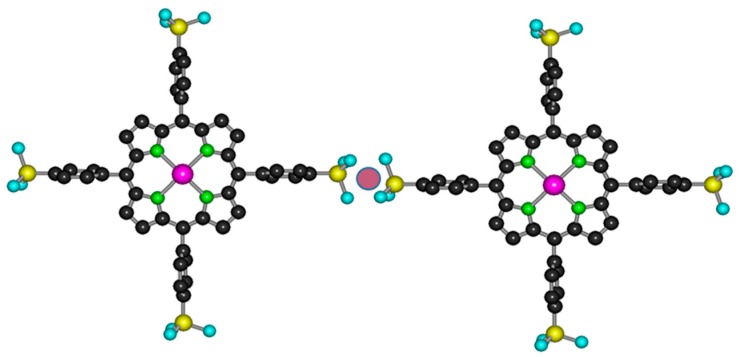
Tail-to-tail connection through the sulfonato groups in lanthanide(III) bisporphyrin. (The hydrogen atoms are omitted for clarity.)

The molar absorption spectrum of these lanthanide(III) bisporphyrins were very similar to those of the corresponding monoporphyrins; further, such analogies were also valid for their emission properties. These strong similarities clearly indicate that the electronic interactions between the porphyrin rings in the lanthanide(III) bisporphyrins studied in this work were very weak—almost negligible. Hence, in accordance with our earlier observation regarding the corresponding La(III), Ce(III), and Nd(III) complexes [15], the monoporphyrin units in the bisporphyrins were connected through a metal bridge between the peripheral sulfonato substituents (i.e., tail-to-tail dimerization, Figure 5).

### 2.3. Photochemistry of Mono- and Bisporphyrins

The out-of-plane position of the metal center in these porphyrin complexes mostly promoted two types of photochemical reactions: (i) dissociation (to free metal ion and porphyrin ligand) without any redox change and (ii) an LMCT process, which generally led to the irreversible cleavage of the macrocycle, producing an open-chain tetrapyrrole derivative (bilindione) [30,52]. This ring-opening process was accompanied by the disappearance of the characteristic porphyrin bands due to the ceasing of the cyclic conjugated double-bond system [20,53]. Although irradiations at both the Soret- and Q-bands resulted in degradation of these lanthanide(III) porphyrins, different intermediates, depending on the excitation wavelength, were detected.

**Figure 6:**
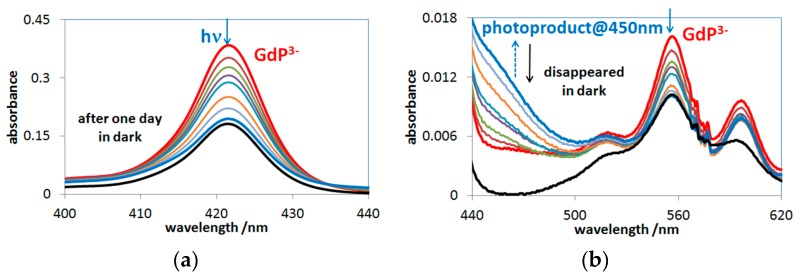
(**a**) Soret- and (**b**) Q-band spectral change during the 45-min Soret-band irradiation of gadolinium(III) porphyrin in the presence of 0.01 M NaAc, 9.2 × 10^−7^ M H_2_TSPP^4−^, 1.0 × 10^−3^ M Gd^3+^, pH ≈ 6, *I*_0_ (421 nm) = 1.2 × 10^−5^ M photons/s, ℓ = 1 cm.

As Figure 6a indicates, Soret-band irradiation led to the gradual decrease of the characteristic bands in both wavelength ranges due to the irreversible ring-cleavage. A careful analysis of the spectral change at longer wavelengths (Figure 6b) revealed the formation of an intermediate, which disappeared in the dark. Such a phenomenon was also observed earlier, in the case of other OOP metalloporphyins (with Bi(III) [19], Hg(II) [40], and Cd(II) [18]). This intermediate, featured by a band at about 450 nm (superimposed on the residual absorbance of the starting porphyrin complexes), may be a radical.

**Figure 7:**
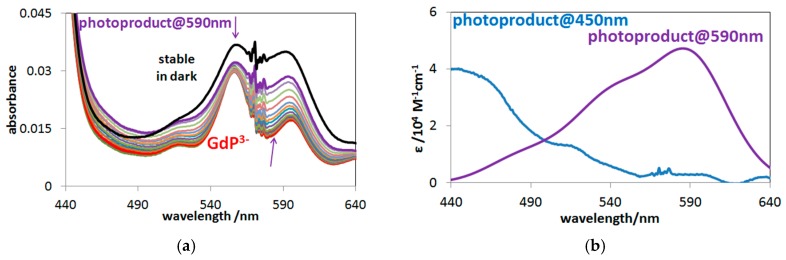
(**a**) Spectral changes during the 45-min photolysis at the Q-maximum (555 nm). (**b**) Molar absorption spectra of the photoproducts.

**Figure 8:**
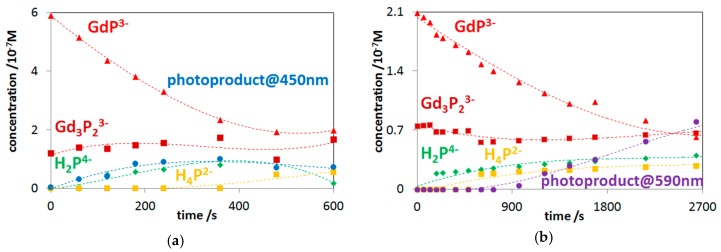
Concentration change of all starting species and the photoproducts at (**a**) Soret-band and (**b**) Q-band irradiations.

In the case of Q-band irradiations (at about 555 nm) of these lanthanide(III) porphyrins, however, deviating from other OOP metalloporphyrins studied so far, a new type of end-product was observed. As Figure 7a shows, this photoproduct gradually accumulated during a 45-min photolysis, and its stability in the dark was well demonstrated by the new absorption band at about 590 nm. This stable product may be a complex formed between a lanthanide(III) ion (in this case, Gd(III)) and the open-chain dioxo-tetrapyrrol photoproduct, due to the favored hard acid-base interaction. Figure 7b shows the estimated molar absorption spectra in the Gd(III) system. Using the individual molar absorbances, the actual concentration versus time data could be calculated for the absorbing species (as described in Section 3.3). The temporal concentration change of the species in the Gd(III)–porphyrin system during the photolyses, at both the Soret- and the Q-band maxima, are shown in Figure 8.

In the systems containing mono- and bisporphyrins in equilibrium, as well as free-base ligands at very low concentrations, all these species were simultaneously excited in the photolysis. Detailed analyses of the spectral changes indicated that, besides the dissociation and photoredox degradation processes, photoinduced transformations between the mono- and bisporphyrin complexes could take place (leading to a steady-state equilibrium, i.e., a photostationary state [37]). Excitation dramatically accelerated these transformations compared with their rates in the dark [38]. Photoinduced ring-oxidation may also take place on the free-base ligand, although with very low quantum yields [49,54,55]. Besides, excitation may increase the rate of metalation and protonation of the free base. Using the molar absorbances of the photoactive species at the excitation wavelength and their concentration versus time data, the individual quantum yields for their photoinduced reactions could be calculated by the procedure described in Section 3.3. Table 3 summarizes the values of these quantum yields for the complexes in the systems studied along with that regarding the photoredox degradation of the free-base porphyrin at Soret-band irradiation. 

In accordance with earlier results [40,49,53], the degradation efficiency of the free-base porphyrin at Soret-band irradiation was very small, that is, two orders of magnitude lower than those regarding the corresponding lanthanide(III) complexes. As these results indicate, the predominant part of the overall quantum yields of the complexes originated from the redox degradation due to the out-of-plane-position of the metal center.

As to the tendency regarding the overall quantum yields, they were unambiguously higher for the bisporphyrins than for their corresponding monomers. This was in accordance with the results obtained on the anionic porphyrins of La(III), Ce(III), and Nd(III) [15]. This phenomenon may be interpreted by the higher probability of utilization of the absorbed photons due to the two metal-porphyrin units, with about the same molar absorbance as that of the corresponding monoporphyrin. The quantum yields for the complexes of the different metal centers did not significantly deviate because their ionic radii and, accordingly, their out-of-plane distances were rather similar. Even so, in the case of the bisporphyrins, a larger ionic radius led to higher overall quantum yield, promoting the demetalation, following the photoinduced electron transfer from the porphyrin ligand to the metal center. These photoinduced features may influence the application of such metalloporphyrins for medical purposes in both therapy and diagnostics.

## 3. Materials and Methods

### 3.1. Materials

Samarium(III), europium(III), and gadolinium(III) were used as chloride salts. Tetrasodium salt of 5,10,15,20-tetrakis(4-sulfonatophenyl)porphyrin (H_2_TSPP^4−^) was applied as a water-soluble porphyrin ligand. Acetic acid and sodium perchlorate were used as ionic strength adjustors. The previous one was also applied for buffers in combination with sodium hydroxide. 1,10-phenanthroline and potassium trisoxalatoferrate(III) were utilized for actinometric measurements. All chemicals were purchased from Sigma-Aldrich Ltd. (Budapest, Hungary) as reagent-grade materials and used without further purification. Argon gas was utilized for deoxygenation of the samples. Doubly distilled water treated with a Millipore Milli-Q system (Millipore SAS., Molsheim, France) was the solvent in this work. The samples for experiments were prepared by using stock solutions of appropriate concentrations. In order to reach the equilibrium state, the solutions were kept in a dark oven at 333 K for at least one day.

### 3.2. Methods 

The UV–Vis spectra were recorded on a Specord S-600 diode-array spectrophotometer (Analytik Jena, Germany). The emission measurements were carried out by using a Fluoromax-4 (Horiba Jobin Yvon, Longjumeau, France) spectrofluorometer equipped with a time-correlated single-photon counting accessory for determination of fluorescence lifetimes. A 393-nm nanoLED was applied for the latter purpose. AMKO LTI photolysis equipment containing a 200-W Xe-Hg lamp and a manually operated monochromator was used for continuous irradiations. The samples were irradiated in glass cuvettes with path lengths of 1 and 5 cm for Soret- and Q-band excitations, respectively. All measurements were carried out at room temperature. Homogenization was ensured by a magnetic stirrer. The irradiated solutions were kept in the dark for one day to check the stability of the photoproducts. Digital evaluations of the experimental data were realized by various procedures using an MS Excel program.

### 3.3. Evaluations

The stability constants and the individual molar absorption spectra of mono- and bisporphyrin complexes in both the Soret- and the Q-range were determined by evaluation of the data regarding the spectrophotometric titrations (described in Section 2.1). For these calculations, the following equations (Equations (1)–(3)) were applied:

(1) $${y\;H}_{2}P^{4^{-}}+{x\;M}^{z+}\Leftrightarrow M_{x}{P_{y}}^{\left(\mathrm{xz}-6y\right)}+{2y\;H}^{+},$$

(2) $$\beta j=\frac{{\beta_{j}}^{\prime }}{{\left[H^{+}\right]}^{2y}}=\frac{\left[M_{x}{P_{y}}^{\left(\mathrm{xz}-6y\right)}\right]}{[H_{2}P^{4^{-}}{]}^{y}[M^{z+}{]}^{x}},$$

(3) $$A_{\lambda }=l\overset{n}{\underset{j=1}{\sum }}\varepsilon_{j\lambda }\beta_{j}\overset{k}{\underset{i=1}{\prod }}{\left[c_{i}\right]}^{\alpha_{\mathrm{ji}}}$$

where *β_j_* designates the formation constant of the jth complex, according to the process described by Equation (1); *A_λ_* and λ are the absorbance and the wavelength, respectively; *l* is the path length (cm); *ε_j__λ_* is the molar absorption coefficient (M^−1^ cm^−1^) at λ wavelength; [c_i_] is the equilibrium concentration of the ith free analyte; and *α_ji_* is its stoichiometric index in the corresponding reaction equation.

Possessing the molar spectra of all the species (*ε_i_*(λ)*)* in the system (both the starting porphyrins and the products), their concentrations (c_i_) could be calculated from the series of the spectra (*A*_obs_(λ,t)) recorded during the irradiations (described in Section 2.3) using Equation (4):

(4) $$A_{\mathrm{obs}}\left(\lambda ,t\right)=\sum A_{i}\left(\lambda ,t\right)=\sum \varepsilon_{i}\left(\lambda \right)\times c_{i}\left(t\right)\times \ell .$$

To calculate the individual quantum yields regarding the photoinduced reactions of each photoactive species (discussed in Section 2.3), the following rate equations (Equations (5)–(9)) were taken into account:

(5) $$d\left\{\mathrm{LnP}\right\}/\mathrm{dt}=v\left(\mathrm{LnP}\;\mathrm{redox}\right)+v\left(\mathrm{LnP}\to H_{2}P\right)+v\left(\mathrm{LnP}\to {\mathrm{Ln}}_{3}P_{2}\right)-v\left({\mathrm{Ln}}_{3}P_{2}\to \mathrm{LnP}\right)-v\left(H_{2}P\to \mathrm{LnP}\right),$$

(6) $$d\left\{{\mathrm{Ln}}_{3}P_{2}\right\}/\mathrm{dt}=v\left({\mathrm{Ln}}_{3}P_{2}\;\mathrm{redox}\right)+v\left({\mathrm{Ln}}_{3}P_{2}\to \mathrm{GdP}\right)+v\left({\mathrm{Ln}}_{3}P_{2}\to H_{2}P\right)-v\left(\mathrm{LnP}\to {\mathrm{Ln}}_{3}P_{2}\right),$$

(7) $$d\left\{H_{2}P\right\}/\mathrm{dt}=v\left(H_{2}P\;\mathrm{redox}\right)+v\left(H_{2}P\to {\mathrm{Ln}}_{x}P_{y}\right)+v\left(H_{2}P\to H_{4}P\right)-v\left({\mathrm{Ln}}_{x}P_{y}\to H_{2}P\right)-v\left(H_{4}P\to H_{2}P\right),$$

(8) $$d\left\{H_{4}P\right\}/\mathrm{dt}=v\left(H_{4}P\;\mathrm{redox}\right)+v\left(H_{4}P\to H_{2}P\right)-v\left(H_{2}P\to H_{4}P\right),$$

(9) $$d\{\mathrm{total}\;\mathrm{porph}.\}/\mathrm{dt}=v(\mathrm{Ln}P\;\mathrm{redox})+v({\mathrm{Ln}}_{3}P_{2}\;\mathrm{redox})+v(H_{2}P\;\mathrm{redox})+v(H_{4}P\;\mathrm{redox}).$$

Using the concentrations of all species absorbing at the excitation wavelength, the amounts of photons absorbed by each of them (*I_i_*) at each experimental point during the irradiation could be calculated by Equation (10):

(10) $$I_{i}=I_{0}\times \left(1-10^{-A_{\mathrm{total}}}\right)\times \frac{1-10^{-A_{i}}}{n-\sum_{j=1}^{n}10^{-A_{j}}}.$$

Finally, taking the values of c_i_ and *I_i_*_,_ calculated at each point of irradiation and *ε_i_*(λ_ir_), the individual quantum yields (*φ_i_* = dc_i_/d*I_i_*) for the photoreactions involved in Equations (5)–(9) could be determined.

## 4. Conclusions

Our results regarding the photoinduced properties of anionic lanthanide(III) porphyrins clearly indicated that in the case of medical applications where excitation of these complexes by visible light occurs (e.g., in photodynamic therapy and luminescence imaging), their photochemical and photophysical behavior ought to be taken into consideration. Since samarium, europium, and gadolinium are in the middle of the lanthanide series, the observed photoinduced features well represent the common (average) characteristics of such Ln(III) complexes.

The concentration-dependent dimerization of the OOP-type monomers through the peripheral sulfonato substituents hardly affects their fluorescence due to the weak interaction between the constituting units, which is advantageous for their usage in diagnostics by luminescence imaging. However, their photolysis reactions (both degradation and dissociation) might be a drawback in their application in diagnostics, although their quantum yields are low. Nevertheless, the photoinduced redox reactions may provide opportunities for their application in photodynamic therapy. Hence, these observations may promote careful selections of appropriate lanthanide(III) porphyrins for various medical applications, considering the aims and conditions of such procedures.

## Figures and Tables

**Table 1:** Formation constants for the mono- and bisporphyrin complexes of Sm(III), Eu(III), and Gd(III).

Complex	SmP^3−^	Sm_3_P_2_^3−^	EuP^3−^	Eu_3_P_2_^3−^	GdP^3−^	Gd_3_P_2_^3−^
Ln^3+^ radius /pm *	107.9		106.6		105.3	
**lgβ_1:1_ and lgβ_3:2_**	4.64	17.47	4.80	17.51	4.94	17.53
**lgβ_1:1_(Ac^−^)**	4.89		5.01		5.11	

* [50].

**Table 2:** Characteristic photophysical data of the investigated lanthanide(III) mono- and bisporphyrins.

Complex	H_2_P^4−^		SmP^3−^	Sm_3_P_2_^3−^	EuP^3−^	Eu_3_P_2_^3−^	GdP^3−^	Gd_3_P_2_^3−^
Ln^3+^ radius/pm *	-		107.9		106.6		105.3	
**λ B(0,0)/nm**	413		421	423	421	423	421	422
**ε B(0,0) /10^5^ M^−1^cm^−1^**	4.66		4.92	4.20	4.92	4.10	4.90	3.98
**λ Q(1,0)/nm**	y 516	x 579	555	557	555	557	555	557
**ε Q(1,0) /10^4^ M^−1^cm^−1^**	y 1.67	x 0.67	1.96	1.74	1.95	1.73	1.92	1.70
ϕ(S_1_-fluo@Q) /10^−2^ **	7.53		4.39	3.09	4.47	2.88	4.15	2.37
Ф(S_1_-fluo@B) /10^−2^ **	5.62		3.03	2.20	2.86	1.92	2.91	1.73
**ϕ(IC S_2_–S_1_)**	0.746		0.69	0.71	0.64	0.67	0.70	0.73
**τ(S_1_) /ns**	10.0		1.93	1.94	1.94	1.95	1.93	1.94
**k_r_(S_1_) /10^6^ s^−1^**	7.5		22.8	16.0	23.1	14.8	21.5	12.3
**k_nr_(S_1_) /10^7^ s^−1^**	9.2		49.6	50.0	49.2	49.8	49.7	50.4

* [50]; ** obtained by excitations at the Q- or B(Soret)-bands.

**Table 3:** Quantum yields for the investigated lanthanide(III) porphyrins, NaClO_4_ = 0.01 M, c(Ln^3+^) = 1.0 × 10^−3^ M and c(H_2_P^4−^) = 1.0 × 10^−6^ M and 0.01 M NaClO_4_.

Complex	H_2_P^4−^	SmP^3−^	Sm_3_P_2_^3−^	EuP^3−^	Eu_3_P_2_^3−^	GdP^3−^	Gd_3_P_2_^3−^
Ln^3+^ radius /pm *	-	107.9		106.6		105.3	
**Ф (S_2_-photochem) /10^−3^**	0.006	0.78	1.67	1.20	1.31	0.86	1.01
**Redox %**	100%	86%	96%	86%	91%	72%	95%
**Dissociation %**	-	11%	3%	7%	4%	15%	4%
**Transformation %**	0% **	3%	1%	7%	5%	13%	1%

* [50] ** In the case of the free-base porphyrin, transformation means protonation and complex formation, but their efficiencies were negligible compared with that of the redox degradation.

## References

[1] Stochel G., Brindell M., Wojciech Macyk W., Zofia Stasicka Z., Szaciłowski K. (2009). Bioinorganic Photochemistry.

[2] Garrett R.H., Grisham C.M. (2010). Biochemistry.

[3] Shriver D., Weller M., Overton T., Rourke J., Armstrong F. (2014). Inorganic Chemistry.

[4] Dąbrowski J.M., Pucelik B., Regiel-Futyra A., Brindell M., Mazuryk O., Kyzioł A., Stochel G., Macyk W., Arnaut L.G. (2016). Engineering of relevant photodynamic processes through structural modifications of metallotetrapyrrolic photosensitizers. Coord. Chem. Rev..

[5] Ladomenou K., Natali M., Iengo E., Charalampidis G., Scandola F., Coutsolelos A.G. (2015). Photochemical hydrogen generation with porphyrin-based systems. Coord. Chem. Rev..

[6] Ertl M., Wöβ E., Knör G. (2015). Antimony porphyrins as red-light powered photocatalysts for solar fuel production from halide solutions in the presence of air. Photochem. Photobiol. Sci..

[7] Knör G. (2015). Recent progress in homogeneous multielectron transfer photocatalysis and artificial photosynthetic solar energy conversion. Coord. Chem. Rev..

[8] Salzl S., Ertl M., Knör G. (2017). Evidence for photosensitised hydrogen production from water in the absence of precious metals, redox-mediators and co-catalysts. Phys. Chem. Chem. Phys..

[9] Radford R.J., Lim M.D., Da Silva R.S., Ford P.C. (2010). Photochemical cleavage of nitrate ion coordinated to a Cr(III) porphyrin. J. Coord. Chem..

[10] Kurtikyan T.S., Hovhannisyan A.A., Gulyan G.M., Ford P.C. (2007). Interaction of Nitrogen Bases with Iron−Porphyrin Nitrito Complexes Fe(Por)(ONO) in Sublimed Solids. Inorg. Chem..

[11] Kurtikyan T.S., Ford P.C. (2008). FTIR and optical spectroscopic studies of the reactions of heme models with nitric oxide and other NOx in porous layered solids. Coord. Chem. Rev..

[12] Fodor M.A., Horváth O., Fodor L., Grampp G., Wankmüller A. (2014). Photophysical and photocatalytic behavior of cobalt(III) 5,10,15,20-tetrakis(1-methylpyridinium-4-yl)porphyrin. Inorg. Chem. Commun..

[13] Fodor M.A., Horváth O., Fodor L., Vazdar K., Grampp G., Wankmüller A. (2016). Photophysical and photochemical properties of manganese complexes with cationic porphyrin ligands: Effects of alkyl substituents and micellar environment. J. Photochem. Photobiol. A Chem..

[14] Major M.M., Horváth O., Fodor M.A., Fodor L., Valicsek Z., Grampp G., Wankmüller A. (2016). Photophysical and photocatalytic behavior of nickel(II) 5,10,15,20-tetrakis(1-methylpyridinium-4-yl)porphyrin. Inorg. Chem. Commun..

[15] Horváth O., Valicsek Z., Fodor M.A., Major M.M., Imran M., Grampp G., Wankmüller A. (2016). Visible light-driven photophysics and photochemistry of water-soluble metalloporphyrins. Coord. Chem. Rev..

[16] Miskolczy Z., Biczók L. (2013). Photochromism of a Merocyanine Dye Bound to Sulfonatocalixarenes: Effect of pH and the Size of Macrocycle on the Kinetics. J. Phys. Chem. B.

[17] Takahashi Y., Fujihara T., Kobayashi N., Nakabayashi S., Miskolczy Z., Biczók L. (2018). Electron transfer kinetics of methylviologen included in 4-sulfonatocalix[n]arenes at glassy carbon electrode; adiabaticity and activation energy. Chem. Phys. Lett..

[18] Valicsek Z., Horváth O., Lendvay G., Kikaš I., Škorić I. (2011). Formation, photophysics, and photochemistry of cadmium(II) complexes with 5,10,15,20-tetrakis(4-sulfonatophenyl)porphyrin and its octabromo derivative: The effects of bromination and the axial hydroxo ligand. J. Photochem. Photobiol. A Chem..

[19] Valicsek Z., Horváth O., Patonay K. (2011). Formation, photophysical and photochemical properties of water-soluble bismuth(III) porphyrins: The role of the charge and structure. J. Photochem. Photobiol. A Chem..

[20] Horváth O., Valicsek Z., Harrach G., Lendvay G., Fodor M.A. (2012). Spectroscopic and photochemical properties of water-soluble metalloporphyrins of distorted structure. Coord. Chem. Rev..

[21] Valicsek Z., Horváth O. (2013). Application of the electronic spectra of porphyrins for analytical purposes: The effects of metal ions and structural distortions. Microchem. J..

[22] Cotton S. (2006). Lanthanide and Actinide Chemistry.

[23] Bünzli J.-C.G. (2017). Rising Stars in Science and Technology: Luminescent Lanthanide Materials. Eur. J. Inorg. Chem..

[24] de Bettencourt-Dias A., Barber P.S., Bauer S. (2012). A Water-Soluble Pybox Derivative and Its Highly Luminescent Lanthanide Ion Complexes. J. Am. Chem. Soc..

[25] Guenet A., Eckes F., Bulach V., Strassert C.A., De Cola L., Hosseini M.W. (2012). Sensitisation of the Near-Infrared Emission of NdIII from the Singlet State of Porphyrins Bearing Four 8-Hydroxyquinolinylamide Chelates. ChemPhysChem.

[26] Semenishyn N.N., Smola S.S., Efryushina N.P., Rusakova N.V. (2015). Spectral and Luminescence Properties of Lanthanide(III) Complexes with Porphyrins and Corroles with Varied Structure. Theor. Exp. Chem..

[27] Faulkner S., Matthews J.L. (2003). Fluorescent Complexes for Biomedical Applications. Comprehensive Coordination Chemistry II.

[28] Bünzli J.-C.G. (2014). Luminescence Bioimaging with Lanthanide Complexes. Luminescence of Lanthanide Ions in Coordination Compounds and Nanomaterials.

[29] Bünzli J.-C.G. (2016). Lanthanide light for biology and medical diagnosis. J. Lumin..

[30] Imran M., Szentgyörgyi C., Eller G., Valicsek Z., Horváth O. (2015). Peculiar photoinduced properties of water-soluble, early lanthanide(III) porphyrins. Inorg. Chem. Commun..

[31] Zhang X., Jiang J., Huang C. (2010). N-Based Rare Earth Complexes. Rare Earth Coordination Chemistry: Fundamentals and Applications.

[32] Bouvet M., Gaudillat P., Suisse J.-M. (2013). Lanthanide macrocyclic complexes: From molecules to materials and from materials to devices. J. Porphyr. Phthalocyanines.

[33] Kiss M.P., Valicsek Z., Horváth O. (2019). Effects of the axial ligands on the formation kinetics of water-soluble cerium(III) porphyrins. Molecules.

[34] Spyroulias G.A., Despotopoulos A.P., Raptopoulou C.P., Terzis A., de Montauzon D., Poilblanc R., Coutsolelos A.G. (2002). Comparative Study of Structure−Properties Relationship for Novel β-Halogenated Lanthanide Porphyrins and Their Nickel and Free Bases Precursors, as a Function of Number and Nature of Halogens Atoms. Inorg. Chem..

[35] Liao M.-S., Watts J.D., Huang M.-J. (2006). DFT/TDDFT Study of Lanthanide III Mono- and Bisporphyrin Complexes. J. Phys. Chem. A.

[36] Zhu X.-J., Zhang T., Zhao S., Wong W.-K., Wong W.-Y. (2011). Synthesis, Structure, and Photophysical Properties of Some Gadolinium(III) Porphyrinate Complexes. Eur. J. Inorg. Chem..

[37] Valicsek Z., Eller G., Horváth O. (2012). Equilibrium, photophysical and photochemical examination of anionic lanthanum(III) mono- and bisporphyrins: The effects of the out-of-plane structure. Dalton Trans..

[38] Kiss M.P., Imran M., Szentgyörgyi C., Valicsek Z., Horváth O. (2014). Peculiarities of the reactions between early lanthanide(III) ions and an anionic porphyrin. Inorg. Chem. Commun..

[39] Wittmer L.L., Holten D. (1996). Photophysics of Lanthanide Triple Decker Porphyrin Sandwich Complexes. Phys. J. Chem..

[40] Valicsek Z., Lendvay G., Horváth O. (2008). Equilibrium, Photophysical, Photochemical, and Quantum Chemical Examination of Anionic Mercury(II) Mono- and Bisporphyrins. J. Phys. Chem. B.

[41] Bünzli J.-C.G. (2010). Lanthanide Luminescence for Biomedical Analyses and Imaging. Chem. Rev..

[42] Khalil G.E., Thompson E.K., Gouterman M., Callis J.B., Dalton L.R., Turro N.J., Jockusch S. (2007). NIR luminescence of gadolinium porphyrin complexes. Chem. Phys. Lett..

[43] Kalota B., Tsvirko M. (2015). Fluorescence and phosphorescence of lutetium(III) and gadolinium(III) porphyrins for the intraratiometric oxygen sensing. Chem. Phys. Lett..

[44] Bulach V., Sguerra F., Hosseini M.W. (2012). Porphyrin lanthanide complexes for NIR emission. Coord. Chem. Rev..

[45] Hu J.Y., Ning Y., Meng Y.S., Zhang J., Wu Z.Y., Gao S., Zhang J.L. (2017). Highly near-IR emissive ytterbium(III) complexes with unprecedented quantum yields. Chem. Sci..

[46] He H. (2014). Near-infrared emitting lanthanide complexes of porphyrin and BODIPY dyes. Coord. Chem. Rev..

[47] Wei C., Ma L., Wei H.B., Liu Z.W., Bian Z.Q., Huang C.H. (2018). Advances in luminescent lanthanide complexes and applications. Sci. China Technol. Sci..

[48] Lipstman S., Muniappan S., George S., Goldberg I. (2007). Framework coordination polymers of tetra(4-carboxyphenyl)porphyrin and lanthanide ions in crystalline solids. Dalton Trans..

[49] Horváth O., Valicsek Z., Vogler A. (2004). Unique photoreactivity of mercury(II) 5,10,15,20-tetrakis(4-sulfonatophenyl)porphyrin. Inorg. Chem. Commun..

[50] Shannon R.D. (1976). Revised effective ionic radii and systematic studies of interatomic distances in halides and chalcogenides. Acta Crystallogr. Sect. A.

[51] Imran M. (2016). Formation, Photophysics and Photochemistry of Water-Soluble Lanthanide(III) Porphyrins. Ph.D. Thesis.

[52] Valicsek Z., Horváth O. (2007). Formation, photophysics and photochemistry of thallium(III) 5,10,15,20-tetrakis(4-sulphonatophenyl)porphyrin: New supports of typical sitting-atop features. J. Photochem. Photobiol. A Chem..

[53] Horváth O., Huszánk R., Valicsek Z., Lendvay G. (2006). Photophysics and photochemistry of kinetically labile, water-soluble porphyrin complexes. Coord. Chem. Rev..

[54] Davila J., Harriman A., Richoux M.-C., Milgrom L.R. (1987). Sterically-hindered zinc porphyrins for solar-energy conversion. J. Chem. Soc. Chem. Commun..

[55] Huszánk R., Lendvay G., Horváth O. (2007). Air-stable, heme-like water-soluble iron(II) porphyrin: In situ preparation and characterization. J. Biol. Inorg. Chem..

